# Burden of infectious diseases in children during the first year after solid organ transplantation

**DOI:** 10.3389/ti.2026.16075

**Published:** 2026-05-22

**Authors:** Nathalie M. Rock, Jaromil Frossard, Christian Vandelden, Giuseppina Sparta, Stefano DiBernardo, Ines Mack, Paloma Parvex, Christian Balmer, Daniela Marx Berger, Christoph Berger, Hassib Chehade, Macé Schuurmans, Sibylle Tschumi, Nicolas J. Mueller, Valérie A. McLin, Arnaud G. L’Huillier

**Affiliations:** 1 Pediatric Gastroenterology, Hepatology and Nutrition Unit, Division of Pediatric Specialties, Department of Pediatrics, Gynecology and Obstetrics, Swiss Pediatric Liver Center, University Hospitals of Geneva, University of Geneva, Geneva, Switzerland; 2 Swiss Transplant Cohort Study (STCS), University Hospital of Basel, Basel, Switzerland; 3 Transplant Infectious Diseases Unit, University Hospitals Geneva, Geneva, Switzerland; 4 Pediatric Nephrology, University Children’s Hospital of Zurich, Zurich, Switzerland; 5 Pediatric Cardiology Service, Department of Woman-Mother-Child, Lausanne University Hospital, Lausanne, Switzerland; 6 Pediatric Infectious Diseases, University Children’s Hospital of Basel, University of Basel, Basel, Switzerland; 7 Pediatric Nephrology, Division of Specialties, Department of Pediatrics, Gynecology and Obstetrics, University Hospitals of Geneva, University of Geneva, Geneva, Switzerland; 8 Pediatric Cardiology, University Children’s Hospital of Zurich, Zurich, Switzerland; 9 Pediatric Nephrology, Children’s Hospital of Eastern Switzerland, St. Gallen, Switzerland; 10 Division of Infectious Diseases and Hospital Epidemiology, University Hospital Zurich, Zürich, Switzerland; 11 Pediatric Nephrology Service, Department of Woman-Mother-Child, Lausanne University Hospital, Lausanne, Switzerland; 12 Pulmonology, University Hospital Zurich, Zurich, Switzerland; 13 Division of Pediatric Nephrology, Department of Pediatrics, Inselspital, Bern University Hospital, University of Bern, Bern, Switzerland; 14 Pediatric Infectious Diseases Unit, Division of General Pediatrics, Department of Pediatrics, Gynecology and Obstetrics, University Hospitals of Geneva, University of Geneva, Geneva, Switzerland

**Keywords:** immunosuppression, infection, opportunistic infection, pediatric, solid organ transplant (SOT), children transplantation, prophylaxis, PTLD

## Abstract

Infections are a leading cause of morbidity and mortality in pediatric solid organ transplant recipients (SOT). Comprehensive data in this population is limited. We included pediatric SOT from the Swiss national cohort aged 0–18 years prospectively from 2008 to 2022. Using standardized definitions, all clinically relevant infections during the first year after transplant were analyzed. Associations with age, organ type, and rejection episodes were assessed. A total of 285 pediatric SOT were included, with kidney (41%) and liver (37%) transplants being the most common. During the first-year post-transplant, 53% (151/285) of patients experienced at least one infection, totaling 360. The overall incidence was 1.36 infection/person/year. Viral infections predominated (53%), followed by bacterial (41%) and fungal infections (6%). Patients receiving liver and lung transplants had higher infection rates (1.91 and 2.53 per person-year, respectively). In multivariate analysis type of transplant and male sex remained associated with increased risk of infection. Viral infections were overrepresented in younger recipients, while bacterial infections were most frequent in the first 3 months post-transplant. Pediatric SOT recipients face a substantial burden of infection. This underscores the need for specific prevention, early recognition, and coordinated management strategies to reduce infection-related morbidity.

## Graphical Abstract

**Figure:**
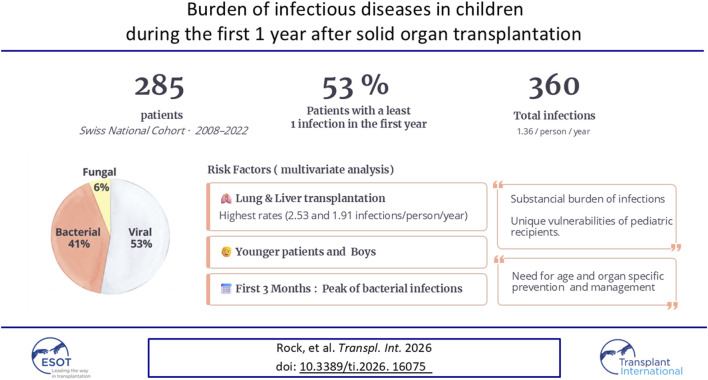

## Introduction

Infections are not only a major cause of short- and long-term morbidity and mortality after solid organ transplantation (SOT) [1, 2], but also an important cause of hospital admission and graft failure [3–5]. The timeline of infections in the first year following SOT was first published more than 25 years ago: at this point, three different periods (0–1, 1–6 and >6 months) associated with different infectious risks were described [6]. A recent analysis of the Swiss Transplant Cohort Study (STCS) highlighted the substantial burden of infections in adult SOT recipients, with 55% experiencing at least one event during the first post-transplant year, predominantly bacterial [7]. However, these results cannot be directly extrapolated to children, whose susceptibility to infection is shaped by developmental and environmental factors: (1) immune system immaturity, (2) limited prior pathogen exposure and adaptive immunity, (3) possible incomplete immunization, and (4) higher exposure in daycare and school settings. In children, infection remains the most common cause of early death after SOT and continues to affect the quality of life for several years thereafter [2, 3, 5, 6]. To the best of our knowledge, no studies have systematically described the incidence and types of infectious complications in a large cohort of pediatric SOT recipients. Reports have generally focused on a single pathogen or on infections in a single organ transplant population. In a cohort of more than 2000 pediatric liver transplant recipients, more than half presented a serious post-transplant infection [8]. Similarly, infection is deemed responsible of 20% of deaths after pediatric kidney transplantation [9]. Therefore, a comprehensive multicenter analysis of post–pediatric SOT infections across various organ types can offer valuable insight into the overall infection burden and facilitate comparisons between transplant groups.

This study aimed to characterize infection incidence during the first post-transplant year in pediatric SOT recipients within the STCS nationwide cohort and to identify associations between infection, age, organ type, and rejection episodes [10].

## Materials and methods

### Study design

This is a prospective study nested in the cumulative recorded data of the STCS. All solid organ transplant recipients (SOT) of all ages transplanted in our country are prospectively enrolled within the STCS. The database, designed as a patient-case system, longitudinally captures organ- and patient-specific data, thus providing an ideal source of unbiased data. Clinical and laboratory information are collected at SOT, 6 and 12 months, and annually thereafter. At each visit, an infectious disease form is completed by an infectious disease physician. Regular monitoring and in-depth audits through review process of randomly selected patients are performed by the STCS Central Data Center.

Ethical approval was obtained from all participating centers. Written informed consent was provided by patients or their legal guardians to participate to the STCS registry, and the study was approved by the scientific committee The present analysis was approved by the STCS Scientific Committee.

### Patients

All SOT (kidney, heart, liver, lung, multi-organ transplant recipients) fulfilling the following criteria were included in the current study: (1) age <18 years at time of SOT; (2) enrolled in the STCS between the start of the registry (01.05.2008) and 31.12.2022, allowing at least 1 year of follow-up. Patients were censored at the last complete infectious disease form, loss of follow-up, new transplantation, graft-loss or after 1 year of follow-up, whatever came first.

### Definitions

#### Infections

Inpatient and outpatient infections were identified by pediatric infectious diseases physicians with experience in transplant infectious diseases, using electronic hospital records and external documentation, according to prespecified rigorous and standardized definitions developed by the STCS infectious diseases working group [7], and based on guidelines and recommendations from the American Society of Transplantation and the European Conference on Infection in Leukemia [11, 12]. Clinically relevant infectious disease events (IDEs) included proven bacterial, probable/proven fungal, and probable/proven viral infections or syndromes; these are hereafter referred to as “infections.” Given the absence of a consensus definition for opportunistic pathogens, we used a list validated by the STCS infectious disease working group, including CMV, EBV, VZV, HHV6, HHV8, adenovirus, BK polyomavirus, *Aspergillus* spp., *Pneumocystis, Zygomycetes, Microsporidia*, *Exophiala*, *Malassezia, Scedosporium, Trichophyton, Alternaria* and *Fusarium* spp., *Toxoplasma gondii, Cryptosporidium* spp., *Giardia lamblia*, *Leishmania* spp., *Strongyloides stercorales*, Isospora belli, N*ocardia* spp., *Listeria monocytogenes*, *Legionella* spp., non-tuberculous mycobacteria.

#### Treatments

The immunosuppressive drugs were recorded at time of the transplantation (−40 days, +14 days). Antimicrobial prophylactic drugs are reported from the day of transplantation to the end of follow-up, they were managed according to institution-specific guidelines based on international recommendations. In absence of exact day for treatment initiation, the first day of the month was imputed. Individual vaccination data were not available in the STCS registry at the time of analysis. Although vaccination policies varied slightly between centers, most followed the Swiss Federal Vaccination Commission schedule [13], with serology-based catch-up vaccination in some centers [14].

#### EBV and CMV risk stratification

The EBV and CMV risk stratification was based on donor (D) and recipient (R) serostatus as follows: high risk: D+/R-; low or intermediate risk: D+/R+, D-/R- or D-/R+.

#### Compatibility and rejection

ABO incompatibility in liver transplantation and donor type in kidney transplantation were recorded, based on the rationale that ABO incompatibility and deceased donor transplantation in kidney recipients are associated with increased immunosuppression. HLA compatibility was not available in the data base. Rejection was defined as biopsy-proven rejection. Management followed organ-specific, center-dependent protocols. The association between infection and rejection was assessed in two directions: rejection occurring within 30 days after infection, considering the potential impact of infections (e.g., CMV) or reduction in immunosuppression; and infection occurring within 90 days after rejection, reflecting the increased infectious risk associated with intensified immunosuppressive therapy.

### Statistical analyses

Baseline characteristics were summarized as counts (percentages) for categorical variables and medians (interquartile range) for continuous variables. Cumulative incidence of first infection was estimated using competing risk methods, and incidence rates expressed per patient-year. Death, graft loss, and re-transplantation were considered as competing events. Incidence rates were calculated accounting for follow-up time using a Poisson generalized linear model, with the log of follow-up time included as an offset. Multivariable generalized linear and cause-specific hazard models identified risk factors adjusted for relevant variables. Detailed methods are available in Supplementary Material 1 [15–19].

## Results

### Baseline characteristics

Out of 8267 adult and pediatric patients transplanted in Switzerland during the study period and included in the STCS, 285 patients met the inclusion criteria and were included in the dataset (Supplementary Figure 1). The baseline characteristics of the cohort are presented in Table 1. Kidney and liver transplantation were the most common, representing 41% (116/285) and 37% (107/285) of all SOT. Liver transplant recipients were the youngest, with a median age at transplant of 1.7 years (IQR 0.8–9.7) whereas lung transplant recipients were the oldest with a median age 15.0 years (IQR 14.0–17.3). Three patients received combined liver-kidney transplants.

**TABLE 1:** Baseline characteristics of pediatric organ transplant recipients included in the study.

Transplanted organ Number of patients	Kidney n = 116	Liver n = 107	Lung n = 14	Heart n = 45	Combined n = 3	Overall n = 285
Recipient age at SOT (yrs), median (IQR)	12.4 (5.7–16.4)	1.7 (0.8–9.7)	15.0 (14.0–17.3)	7.8 (1.5–14.1)	10.4 (2.4–11.6)	8.6 (1.8–15.1)
Recipient age category, n (%)						
<1 year	8 (6.9)	56 (52.3)	0 (0.0)	12 (26.7)	0 (0.0)	76 (26.7)
1–4.99 years	22 (19.0)	16 (15.0)	1 (7.1)	8 (17.8)	1 (33.3)	48 (16.8)
5–11.99 years	30 (25.9)	14 (13.1)	2 (14.3)	11 (24.4)	2 (66.7)	59 (20.7)
12–17.99 years	56 (48.3)	21 (19.6)	11 (78.6)	14 (31.1)	0 (0.0)	102 (35.8)
Recipient sex (male), n (%)	67 (57.8)	61 (57.0)	6 (42.9)	14 (31.1)	1 (33.3)	149 (52.3)
Previous transplant history, n (%)	7 (6.0)	2 (1.9)	0 (0.0)	0 (0.0)	1 (33.3)	10 (3.5)
Donor age at donation (yrs), median (IQR)	38.5 (25.0–45.0)	22.0 (12.0–43.0)	34.0 (25.0–41.0)	9.0 (2.0–24.0)	26.0 (10.0–44.0)	32.0 (16–43.0)
Donor sex (male), n (%)	67 (57.8)	58 (54.2)	7 (50.0)	16 (35.6)	1 (33.3)	149 (52.3)
CMV risk stratification, n (%)						
High risk	44 (38.3)	14 (13.6)	4 (28.6)	15 (33.3)	0 (0.0)	77 (27.5)
Low or intermediate risk	71 (61.7)	89 (86.4)	10 (71.4)	30 (66.7)	3 (100.0)	203 (72.5)
EBV risk stratification, n (%)						
High risk	42 (36.5)	40 (38.5)	2 (16.7)	13 (29.5)	1 (33.3)	98 (35.3)
Low or intermediate risk	73 (63.5)	64 (61.5)	10 (83.3)	31 (70.5)	2 (66.7)	180 (64.7)
Induction drugs						
Basiliximab, n (%)	71 (61.2)	92 (86.0)	12 (85.7)	13 (28.9)	3 (100.0)	191 (67.0)
ATG, n (%)	2 (1.7)	1 (0.9)	0 (0.0)	30 (66.7)	2 (66.7)	35 (12.3)
Immunosuppressive drugs^a^						
MMF n (%)	113 (97.4)	15 (14.0)	14 (100.0)	40 (88.9)	3 (100.0)	185 (64.9)
TAC, n (%)	79 (68.1)	106 (99.1)	5 (35.7)	24 (53.3)	1 (33.3)	215 (75.4)
CsA, n (%)	40 (34.5)	3 (2.8)	10 (71.4)	20 (44.4)	1 (33.3)	74 (26.0)
GC, n (%)	113 (97.4)	78 (72.9)	14 (100.0)	44 (97.8)	3 (100.0)	252 (88.4)
Other, n (%)	4 (3.4)	1 (0.9)	1 (7.1)	7 (15.6)	1 (0.0)	14 (5)
Outcome^b^, n (%)						
Death (1 year)	0 (0.0)	5 (4.7)	2 (14.3)	2 (4.4)	2 (66.7)	11 (3.9)
Dropout (1 year)	1 (0.9)	0 (0.0)	0 (0.0)	0 (0.0)	0 (0.0)	1 (0.4)
End follow-up (<1 year)	2 (1.7)	2 (1.9)	0 (0.0)	2 (4.4)	0 (0.0)	6 (2.1)
Graft loss, PNF (1 year)	3 (2.6)	3 (2.8)	2 (14.3)	0 (0.0)	0 (0.0)	8 (2.8)
Number of infections (1year), n (%)						
0	67 (57.8)	36 (33.6)	5 (35.7)	24 (53.3)	2 (66.7)	134 (47.0)
1	28 (24.1)	23 (21.5)	3 (21.4)	12 (26.7)	1 (33.3)	67 (23.5)
2	9 (7.8)	18 (16.8)	3 (21.4)	8 (17.8)	0 (0.0)	38 (13.3)
3+	12 (10.3)	30 (28.0)	3 (21.4)	1 (2.2)	0 (0.0)	46 (16.1)
Infections incidence rate^b^	1.00 (0.82; 1.19)	1.91 (1.65; 2.19)	2.53 (1.68; 3.62)	0.74 (0.51; 1.03)	-	1.36 (1.22; 1.51)

SOT, solid organ transplant; ATG, anti-thymocyte globulin; IQR, interquartile range; CMV, cytomegalovirus; EBV, Epstein-Barr virus; MMF, mycophenolate mofetil; TAC, tacrolimus; CsA, cyclosporine; GC, glucocorticoids.

^a^ All immunosuppressive drugs recorded (within 14 days after and 30 days before transplantation).

^b^ Censored after graft losses, infections incidence rate : infection/patient/year PNF: Primary non Function.

CMV/EBV risk stratification based on donor (D) and recipient (R) serostatus: high risk: D+/R-; low or intermediate risk: D+/R+, D-/R- or D-/R+.

During the study follow-up, twelve patients died (4%). Graft loss occurred in eight (3%), leading to retransplantation in six (Table 1).

Immunosuppressive regimens per organ at baseline and throughout the first year are detailed in Table 1 and in Supplementary Figure 2.

Prophylactic strategies are shown in Supplementary Figure 3. Prophylaxis against *Pneumocystis jirovecii* was given to 94% (267/285) for a median of 348 days (IQR 186–261). Additionally, 73% (207/285) received antiviral prophylaxis for a median of 185 days (IQR 116–288), mainly (val)ganciclovir in 68% (194/285). Antifungal prophylaxis was used in 17% (48/285), mostly in lung or combined transplant recipients.

### Infectious diseases events

Amongst the 285 patients included in the study, 53% (n = 151) presented at least one infection during the first year after SOT. A total of 360 infections were documented; 16% of patients had ≥3 events (Table 1) and 29% at least 2 infectious events. The infection incidence rate was 1.36 infection/patients/year (95% confidence interval [CI]: 1.22–1.51).

**FIGURE 1:**
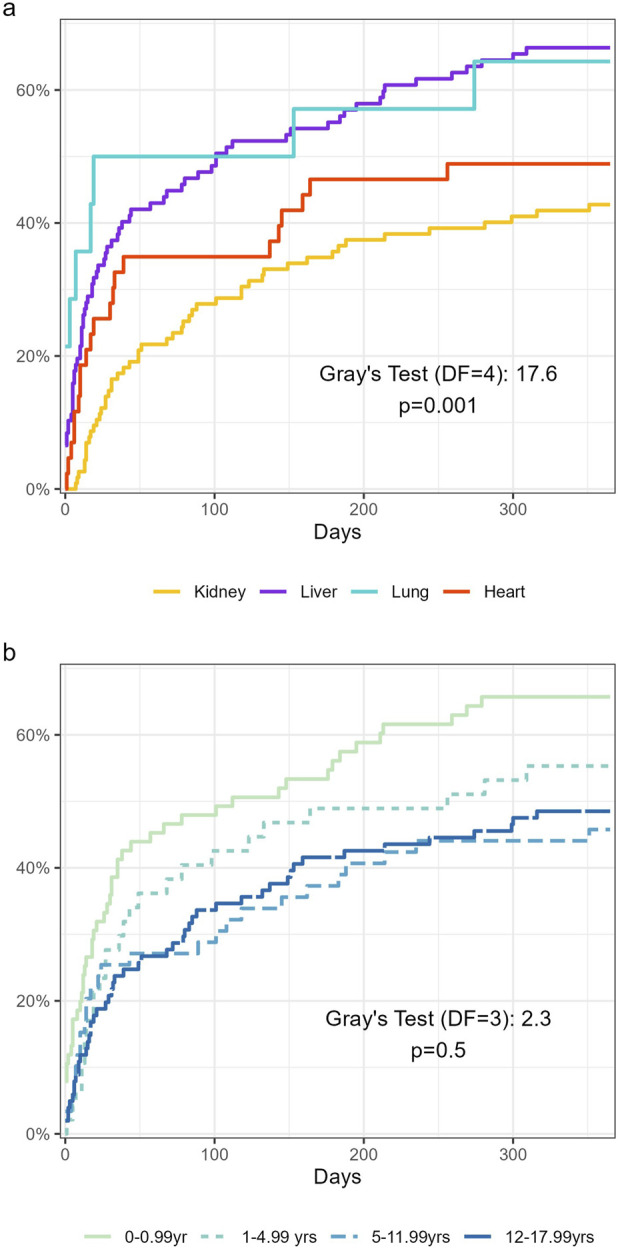
Breakdown of infections between **(a)** organs **(b)** age groups. All organs include 1 infection from combined transplant not displayed in the Panel **(a)**.

**FIGURE 2:**
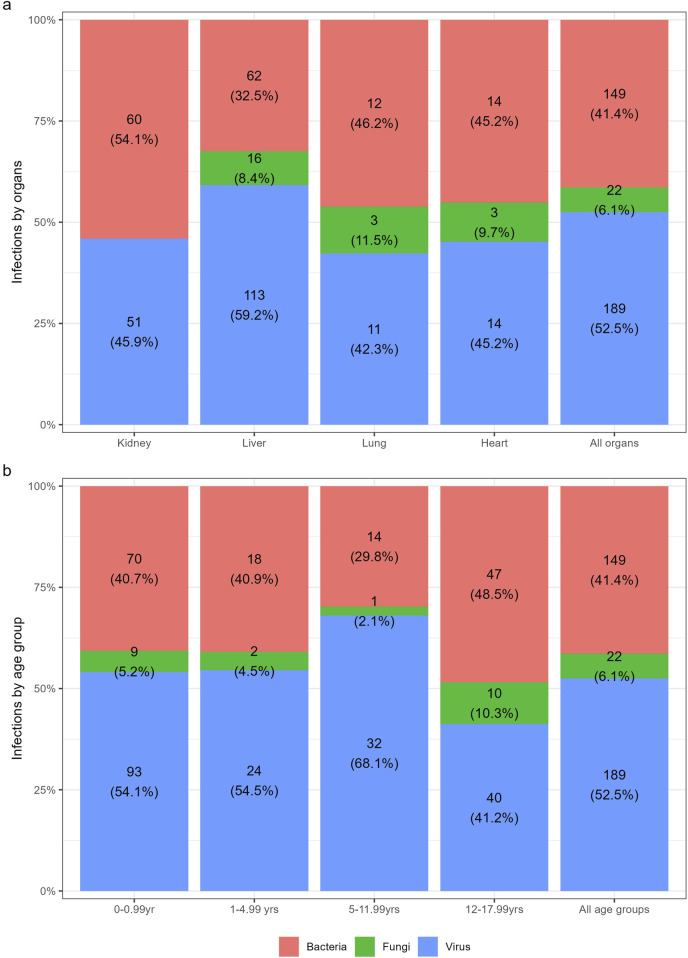
Cumulative incidence of infections between **(a)** organs **(b)** age groups. All organs include 1 infection from combined transplant taken in consideration for the gray test.

The breakdown of infections between organs is depicted in Figure 1a. Patients after lung and liver transplantation exhibited the highest incidence of infection (2.53 [95%CI: 1.68–3.62] and 1.91 [95%CI: 1.61–2.19], respectively) and heart transplants the lowest (0.74 [95%CI: 0.51–1.03]). The cumulative incidence of infection significantly differed between organs (p = 0.001) (Figure 2a). Among, the three combined transplants, one gastrointestinal infection with multiple bacteria was observed.

The distribution of infection among age group is presented in Figure 1b. Sixty-four percent of patients <1 year of age presented with at least one infection during the first year after SOT (49/76), compared to 54% (26/48), 46% (27/59) and 48% (49/102) for those between 1 and <5, between 5 and <12, and between 12 and < 18 years, respectively. Amongst patients aged <1 year at time of transplant, the infection incidence rate was 2.41 (95% CI: 2.07–2.79), compared to 0.96 (95% CI: 0.70–1.27), 0.84 (95% CI: 0.62–1.11) and 1.06 (95% CI: 0.86–1.28) in those between 1 and <5, between 5 and <12, and between 12 and <18 years, respectively. The cumulative incidence of infection is described in Figure 2b.

Forty-two patients with living-donor kidney transplants experienced 53 infections (1.26 infections per patient), compared with 58 infections among 74 patients with deceased-donor transplants (0.78 infections per patient).

### Burden of infectious diseases events

Amongst the 360 infections; 53% were caused by viruses (n = 189), followed by bacteria (41% n = 149]) and fungi (6% [n = 22]) (Figure 1).

#### Viral infections

**FIGURE 3:**
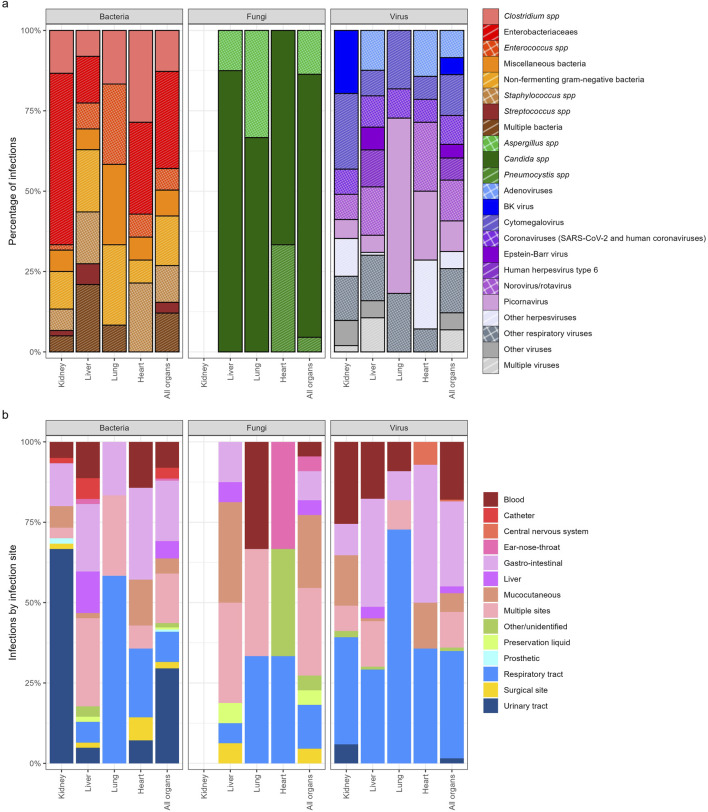
**(a)** Causal Infectious agent in bacterial, fungal and viral infections by organ. **(b)** Infectious site of bacterial, fungal and viral infections by organ.

Amongst the 189 viral infections, proven infections were documented in 110 (58%), probable infections in 69 (37%), and viral syndromes in 10 (5%) cases. Respiratory viruses predominated, followed by CMV (13%, 24/189) of all viral infections (Figure 3a; Supplementary Table 1a). Sixty percent of viral infections occurred in liver transplant recipients (113/189). Respiratory and gastrointestinal systems were most affected.

Around half of the viral infections occurred in children <1 year at SOT (49% [93/189]) (Supplementary Table 1b). Twelve EBV infections occurred in 10 patients, half of whom were stratified as EBV high risk at transplantation. Four patients developed post-transplant lymphoproliferative disorder (PTLD) between 0 and 160 days after EBV infection. Additionally, 27 CMV infections were recorded in 25 patients, including 15 stratified as high risk at transplant. Most CMV episodes (19/27) occurred in the absence of antiviral prophylaxis, whereas 30% (8/27) were breakthrough episodes.

#### Bacterial infections

Amongst the 149 bacterial infections, *E. coli* was the most commonly detected pathogen in 17% of cases (25/149), followed by *Clostridium* spp. (13% [19/149]) and *Pseudomonas* spp. (13% [19/149]) (Figure 3a; Supplementary Table 1a). A total of 45% (68/149) of bacterial infections were caused by Gram-negative organisms. Most cases of bacterial infections occurred in liver (42% [62/149]) and kidney (40% [60/149]) transplant recipients. Around half of the bacterial infections occurred in children <1 year at SOT (47% [70/149]) (Supplementary Tables 1a,b). Urinary tract infections predominated in kidney recipients, and respiratory infections in lung and heart recipients.

Thirteen patients (liver [n = 6], kidney [n = 6]), lung [n = 1] transplant recipients) experienced at least one infection caused by a multi-drug resistant (MDR) bacteria, totaling 19 episodes. Of the 19 episodes of infections caused by MDR bacteria, the most common organisms were MDR *Enterobacter* spp. (n = 7) and *E. coli* spp. (n = 6). Other identified MDR bacteria were *Pseudomonas* spp. (n = 3), *Klebsiella* spp. (n = 2) and *Acinetobacter* spp (n = 1). No carbapenemase-producing Enterobacterales (CPE) or methicillin-resistant *Staphylococcus aureus* (MRSA) were identified.

#### Fungal infections

Amongst the 22 fungal infections, proven infection was documented in 16 (73%), the remaining being documented as probable fungal infection. The most frequently observed pathogen was *Candida* albicans, representing 59% (13/22) of fungal infections (Figure 3a; Supplementary Tables 1a,b). Death occurred in 3/22 cases (13.6%) 2 candidas and one other fungi, none of which were receiving antifungal prophylaxis, while 4/22 infections (18.2%) occurred despite prophylaxis.

#### Vaccine-preventable pathogens

Among 360 infections, 27 were attributed to vaccine-preventable pathogens (6%). Of these, 12 infections were caused by rotavirus, seven by influenza, six by varicella-zoster virus, one by *Haemophilus* influenzae type b and one by hepatitis B virus (donor-derived).

#### Opportunistic pathogens

Among 360 infections, 83 (23%) were secondary to opportunistic pathogens. These pathogens were mostly viruses (93% [77/83]), such as CMV (n = 24), adenoviruses (n = 16), HHV6 (n = 13), and BK virus (n = 10), followed by EBV (n = 8) and VZV n = 6). Other opportunistic pathogens were rare (*Aspergillus* spp [n = 3], non-tuberculosis mycobacteria ([n = 2]; *Pneumocystis* spp [n = 1]).

#### Site of infections

Most occurred in abdominal sites (liver, gastrointestinal site and urinary tract - 39%,140/360), followed by respiratory infections (22%, 80/360) (Figures 3a,b; Supplementary Table 2).

#### Timeline of infections

**FIGURE 4:**
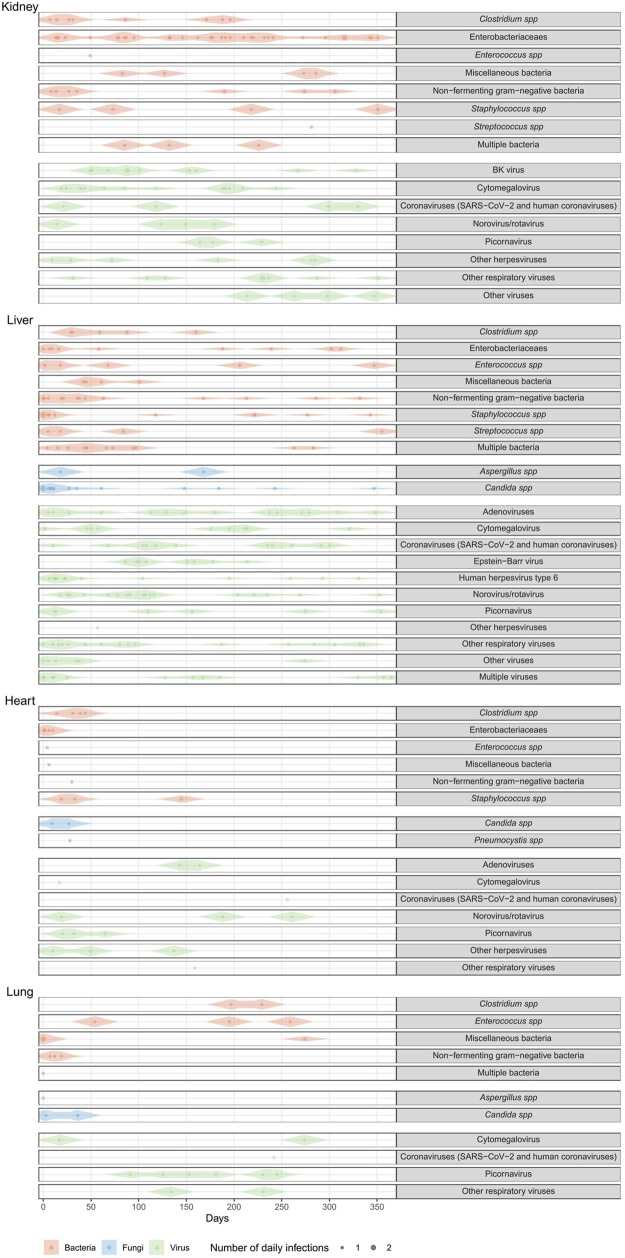
Violin representation of infections over time during the first year after transplant.

The burden of infections over time is presented by organ in Figure 4. Bacterial infections predominated in the first 3 months (56%, (84/149)), mainly *Clostridium* spp, mixed species, and *Pseudomonas* spp. *E. coli* infections occurred throughout the year (Supplementary Table 3).

Viral infections remained steady; EBV appeared mainly between 3 and 6 months, CMV throughout, and gastrointestinal viruses year-round. Most fungal infections occurred in the first month, mainly *Candida* spp (Supplementary Table 3).

#### Infections by organ

Regarding bacterial infections, in the kidney, Gram-negative bacteria predominated, with *E. coli* accounting for 17 cases (28.3%), followed by *Klebsiella spp.* (8; 13.3%) and *Pseudomonas spp.* (8; 13.3%), while gram-positive organisms remained less frequent. In the liver, infections were more evenly distributed, with 22/62 (35.5%) Gram-negative and 25/62 (40.3%) Gram-positive bacteria, alongside 13/62 (21%) polymicrobial cases. In the lung, the distribution was heterogeneous, with notable proportions of *Enterococcus spp.* (3/12; 25%) and mycobacteria (2/12; 16.7%), and no clear predominance of Gram-negative organisms. In the heart, Gram-positive bacteria dominated (5/7; 71.4%), mainly *Clostridium spp.* (2/7; 28.6%) and staphylococci (1/7; 14.3%), whereas Gram-negative bacteria were rare (1/7; 14.3%) and polymicrobial infections uncommon.

Regarding viruses, in the kidney, infections were mainly due to BK virus (10/51; 19.6%) and cytomegalovirus (12/51; 23.5%), whereas the liver showed a broader distribution including adenovirus (14/113; 12.4%), HHV-6 (13/113; 11.5%), coronaviruses (11/113; 9.7%), and 12/113 (10.6%) multiple viral infections. In the lung, picornaviruses predominated (6/11; 54.5%), while the heart displayed a low and heterogeneous viral distribution without a dominant pathogen.

Four patients liver-transplanted of a ABO incompatibly context experienced 9 infections (2.25 infections per patient). Forty-two patients with living-donor kidney transplants experienced 53 infections (1.26 infections per patient), compared with 58 infections among 74 patients with deceased-donor transplants (0.78 infections per patient).

### Management of infections

Among the 360 infections, information regarding inpatient vs. outpatient management was available in 290 (81%). Among those, 58% (169/290) required hospitalization for management while 42% (122/290) were managed on an outpatient basis. Ninety-nine percent (146/148) of bacterial infections were treated by antibacterial therapy. Antiviral therapy was administered in 30% (55/185) of viral infections. All fungal infections were treated with antifungals. Additionally, a reduction of immunosuppression was reported in 10% of infections for which information was available (34/335). Of these cases, 29 involved viral infections (11 CMV; 6 EBV; 6 BK virus).

Among the 11 patients who died, seven had an active infection at the time of death. Five deaths were attributed to multiorgan failure secondary to infection, including three due to invasive fungal infections (one Aspergillus spp with multisystemic involvement and two *Candida* spp affecting the respiratory and gastrointestinal tract) and two due to bacterial sepsis (Herbaspirillum spp. in blood and *Bacteroides fragilis* from a gastrointestinal source). The remaining two patients died from non-infectious causes (cerebrovascular disease and postoperative hemorrhage), both associated with concurrent infection.

### Risk factors for infections

Demographics of patients with (n = 151) and without infections (n = 134) are compared in Table 2. In a univariate model, liver and lung transplantation were associated with an increased infection incidence rate ratio (IRR) (2.16; p < 0.001 and 2.99; p < 0.007, respectively). A younger recipient age as well as male sex were also associated with an increased IRR (0.96; p = 0.009 and 1.54; p = 0.016, respectively).

**TABLE 2:** Comparison of patients with and without infection.

Characteristic Number of patients	Infections n = 151	No infections n = 134	Univariate IRR (95% CI), p-value	Multivariate IRR (95% CI), p-value^a^
Transplanted organ, n (%)				
Kidney	49 (32.5)	67 (50.0)	​	​
Liver	71 (47.0)	36 (26.9)	2.16 (1.47–3.18), p < 0.001	2.14 (1.38–3.34) p < 0.001
Lung	9 (6.0)	5 (3.7)	2.99 (1.34–7.15) p = 0.007	4.09 (1.79–10.1) p < 0.001
Heart	21 (13.9)	24 (17.9)	0.74 (0.42–1.29) p = 0.29	0.87 (0.49–1.53) p = 0.62
Combined	1 (0.7)	2 (1.5)	​	​
Recipient age at transplant (yrs), median (IQR)	6.3 (1.4–14.3)	10.5 (2.9–15.6)	0.96 (0.94–0.99) p = 0.009	0.99 (0.95–1.02) p = 0.39
Recipient sex (male), n (%)	84 (55.6)	65 (48.5)	1.54 (1.07–2.21) p = 0.016	1.44 (1.01–2.06) p = 0.038
Previous transplant history, n (%)	4 (2.6)	6 (4.5)	​	​
CMV risk stratification, n (%)				
High risk	44 (29.7)	33 (25.0)	0.85 (0.57–1.28) p = 0.43	1.04 (0.69–1.56) p = 0.86
Low or intermediate risk	104 (70.3)	99 (75.0)	​	​
Unknown	3 (2.0)	2 (1.5)	​	​
EBV risk stratification, n (%)				
High risk	57 (38.0)	41 (32.0)	1.19 (0.83–1.73) p = 0.43	1.22 (0.83–1.80) p = 0.29
Low or intermediate risk	93 (62.0)	87 (68.0)	​	​
Unknown	1 (0.7)	6 (4.5)	​	​
Inductions drugs^b^				
Basiliximab, n (%)	113 (74.8)	78 (58.2)	1.91 (1.28–2.85) p = 0.001	1.59 (0.98–2.60) p = 0.057
ATG, n (%)	12 (7.9)	23 (17.2)	0.35 (0.18–0.67) p = 0.002	1.05 (0.37–2.94) p = 0.92
Immunosuppressive drugs				
MMF, n (%)	84 (55.6)	101 (75.4)	0.52 (0.36–0.75) p = 0.001	0.79 (0.37–1.71) p = 0.51
TAC, n (%)	107 (70.9)	108 (80.6)	0.86 (0.57–1.30) p = 0.48	0.83 (0.37–1.81) p = 0.64
CsA, n (%)	49 (32.5)	25 (18.7)	1.22 (0.82–1.84) p = 0.32	1.66 (0.76–3.66) p = 0.21
GC, n (%)	129 (85.4)	123 (91.8)	0.71 (0.41–1.18) p = 0.19	0.99 (0.58–1.67) p = 0.21
Other, n (%)	7 (4.6)	6 (4.5)	0.46 (0.18–1.20) p = 0.11	0.85 (0.31–2.28) p = 0.73
Number of different IS, median (IQR)	3 (2–3)	3 (2–3)	0.7 (0.56, 0.88), p = 0.002	0.98 (0.72, 1.34) p = 0.9

^a^ IRR derived from negative binomial generalized linear model. Effects highlighted in gray are adjusted for Organs, Sex, Age, Gender, CMV Serology and EBV Serology. Effects highlighted in blue are additionally adjusted for Basiliximab, ATG, MMF/EC-MPA, TAC, CsA, GC, Other IS. Effect highlighted in green are adjusted for the set RF1, Basiliximab and ATG.

^b^ All immunosuppressive drugs recorded (within 14 days after and 30 days before transplantation).

IRR: Incidence rate ratio; IQR: interquartile range; yrs: years; CMV: Cytomegalovirus; EBV: Epstein-Barr virus, ATG: anti-thymocyte globulin;

IS, Immunosuppressor. MMF, Mycophenolate mofetil. TAC, Tacrolimus. CsA, cyclosporine A. GC, Glucocorticoid.

CMV/EBV risk stratification based on donor (D) and recipient (R) serostatus: high risk: D+/R-; low or intermediate risk: D+/R+, D-/R- or D-/R+.

In a multivariate model, only liver and lung transplantation as well as male sex remained associated with an increased infection incidence rate ratio (2.14; p < 0.001, 4.09; p < 0.001, 1.44; p = 0.038 respectively). (Table 2). No associations were found with immunosuppressive regimen or number of drugs (Table 2). Organ specific analyses did not identify any additional risk factors for infections beyond male sex (Supplementary Figure 5).

#### Association with rejection

During the follow-up period, 155 episodes of biopsy-proven rejections were observed with a median time of 51 days after transplantation (IQR 18–166) in 85 patients. Fifteen percent (24/155) episodes of rejection occurred within 30 days after an infection (Supplementary Table 4). In the multivariate model, no significant risk of rejection was found in the 30 days following any infection (HR 1.08; 95% Cl 0.57–2.06; p = 0.800).

Twenty-five episodes of rejection were followed by at least one infection within 90 days, totaling 43 infections (one infection in 16 cases, >1 infection in 9 cases) (Supplementary Table 4). In the multivariate model, rejection was not identified as a risk factor of infection (HR 1.08; 95% Cl 0.58–1.99; p = 0.800). Supplementary Figure 4 displays the timeline between rejection and infections by organ and by patient.

## Discussion

This nationwide study of pediatric SOT during the first year after transplantation highlights several findings: (1) more than half of patients experienced at least one infectious event; (2) incidence of infection was highest in younger recipients; (3) liver and lung transplantation, as well as male sex were independently associated with an increased risk of infections (5) viral infections accounted for the largest proportion of infections; (6) bacterial infections predominated during the first 3 months, with a large proportion due to Gram-negative organisms (6) distinct organ-specific microbiological patterns were observed (7) and the burden of fungal infection was considerable, with 3 of 22 fungal infections resulting in death.

To our knowledge, this is the first comprehensive and standardized characterization of the infectious burden within the first year after pediatric SOT in a nationwide cohort. Most studies have focused on a single type of infection or on severe infections [20, 21], a single type of transplanted organ [8, 22], or data collection was restricted to the immediate early post-SOT period [23]. While organ-specific studies are essential, our objective is complementary: to provide an overall view of the infectious burden across pediatric SOT and enable comparisons between transplant groups, that share underlying immunosuppressive mechanisms. Our data showed that approximately 50% of pediatric SOT recipients presented at least one infection during the first year after transplant, in line with previous reports after pediatric liver transplantation [8]. Notably, one fifth of patients experienced >3 infections. Nearly half of infections required hospitalization. The challenge of invasive diagnostic and therapeutic procedures in children (venous access, biopsies of the infected sites,…), the impact on quality of life, and the economic burden of these infections were not analyzed in this study but are likely to be considerable [24].

At an incidence rate of 1.36 infection, the incidence of infection in our cohort was very similar to adult SOT recipients using the same design, definitions, and study population [7]. However, infection patterns differed substantially between children and adults, supporting the need for age-specific preventive and management strategies.

Infections were more frequent in younger patients, likely to immune immaturity and more frequent behavioural exposure. Young children lack antigen-specific adaptive responses and therefore rely on stronger but less specific innate responses [25, 26]. Lung transplant recipients had the highest incidence of infections, likely attributable to intense immunosuppression, airway exposure to pathogens and impaired mucociliary clearance [27–29]. Liver transplant recipients also had a high incidence of infections, despite a lower immunosuppression [8], and even after adjusting for age. This may reflect the liver immunologic role, cirrhosis-related immune defects [30–35] and the role of basiliximab induction. Male sex remained an independent risk factor, consistent with known sex-based immune differences and possible behavioral factors, including treatment non-adherence [36–47].

More than half of infections were viral, contrasting with the adult study where bacterial infections predominated [7]. Viral infections were even more frequent in younger children and occurred year-round, reflecting transitions from donor-derived and immunosuppression-related risk to community exposure after return to school or daycare. Respiratory and gastrointestinal viruses were most common, mirroring pediatric viral epidemiology [48–50], followed by herpesviruses, reflecting their burden after pediatric SOT [51–54]. These findings emphasize the need for heightened vigilance for viral infections in routine pediatric follow-up, early testing for respiratory pathogens in order to avoid unnecessary prolonged empiric antibiotherapy, importance of immunization in the community for vaccine-preventable diseases immunization and infection control measures such as masks, social distancing and hand hygiene whenever possible in case of sustained viral circulation in schools and daycare.

Bacterial infections predominated in the early months, reflecting intensified immunosuppression and post-operative complications. Like in adult SOT recipients [7], *E. coli* was the most frequent cause of bacterial infections, of which 24% were MDR. [12]. The high prevalence of Gram-negative pathogens in this cohort supports the adaptation of empirical antibacterial therapy toward broader-spectrum coverage in cases of suspected bacterial infection. These findings also support the importance of early postoperative surveillance cultures in febrile patients and emphasize the need for close multidisciplinary coordination between transplant teams and community pediatricians to ensure timely detection and rapid referral for suspected severe infections.

Moreover, our findings reveal clear organ-specific microbiological patterns. Kidney infections were largely driven by enteric Gram-negative bacteria, consistent with a urinary or digestive origin, whereas liver infections displayed a mixed and frequently polymicrobial profile, supporting a biliary or intra-abdominal source [55, 56]. Lung infections were more heterogeneous and included opportunistic pathogens, suggesting a role for host-related factors such as immunosuppression. In contrast, heart infections were predominantly caused by Gram-positive bacteria, reflecting typical endovascular infections associated with skin flora and biofilm formation.

Viral infections also exhibited organ-specific distributions, with latent viruses predominating in the kidney and a broader, more diverse spectrum in the liver, possibly reflecting younger age and primary exposure to common viruses. Lung infections were mainly driven by respiratory viruses, whereas cardiac involvement remained rare and nonspecific.

Although the number of reported fungal infections remained low, the outcome was fatal in 13% of cases. This raises the question of whether the indications for antifungal prophylaxis in SOT recipients, rarely applied in our cohort, should be expanded, and of the clinical situations in which screening for fungal colonization should be considered.

Less than a quarter of infections in our dataset were caused by opportunistic pathogens. This is probably overestimated given the fact that some viral pathogens considered as opportunistic by the STCS infectious diseases working group might indeed be opportunistic in the adult setting, but might simply reflect primary community infection in children (i.e., VZV, adenovirus). Overall, the relatively low burden of opportunistic infections likely reflects effective prophylaxis, underscoring adherence to standardized protocols and ongoing education of families and healthcare providers. Also, only 6% of infections were attributed to vaccine-preventable pathogens. While this may suggest a potential contribution of the pre-transplant immunization to post-transplant protection, this could not be verified in our cohort as individual immunization charts were not available. [14]. These findings nonetheless support the importance of ensuring up to date vaccination before transplantation and post-transplant catch-up when feasible.

No significant association was found between rejection episodes and infections. This may reflect limited power and pathogen-specific differences in the risk of rejection [57]. In this cohort, steroid-free regimens were not associated with a reduced risk of infection. However, this finding may be subject to selection bias, as most patients received steroid-based therapy. Contrary to our initial hypothesis, recipients of deceased-donor kidney transplants did not exhibit a higher infection rate, as the number of infections per patient was greater in the living-donor group.

Limitations of our study include the sample size limiting conclusion of subgroup analyses, possible reporting bias toward severe infections (as 60% required hospitalization), and underreporting of milder community-acquired cases. The absence of a pediatric control group limits comparison with the general population, and the results may not be generalized to other settings with different epidemiology or clinical practices. In addition, detailed data on immunosuppressive drug dosing and levels, treatment of rejection were unavailable restricting analysis of their association with infection. As with all registry-based studies, our findings may also be affected by loss to follow-up, missing or incomplete data, and variability in data reporting across centers.

In conclusion, this study provides a comprehensive overview of the infectious burden in the first year following pediatric SOT. More than half of patients experienced at least one clinically relevant infection, with viral infections predominated highlighting the unique vulnerabilities of pediatric recipients. The higher risk of infection was for liver and lung transplant recipients. These findings underscore the need for age and organ specific prevention and management strategies, including optimized vaccination, early diagnostics, and tailored antimicrobial approaches.

## Data Availability

The raw data supporting the conclusions of this article will be made available by the authors, without undue reservation.

## Group members of Swiss Transplant Cohort Study

Member of the Swiss Transplant Cohort Study: Patrizia Amico, Adrian Bachofner, Vanessa Banz, Sonja Beckmann, Guido Beldi, Christoph Berger, Ekaterine Berishvili, Annalisa Berzigotti, Françoise-Isabelle Binet, Pierre-Yves Bochud, Petra Borner, Sanda Branca, Anne Cairoli, Emmanuelle Catana, Yves Chalandon, Philippe Compagnon, Sabina De Geest, Sophie De Seigneux, Michael Dickenmann, Joëlle Lynn Dreifuss, Thomas Fehr, Sylvie Ferrari-Lacraz, Andreas Flammer, Jaromil Frossard, Déla Golshayan, Nicolas Goossens, Fadi Haidar, Jürg Halter, Christoph Hess, Sven Hillinger, Hans Hirsch, Patricia Hirt, Linard Hoessly, Uyen Huynh-Do, Franz Immer, Nina Khanna, Michael Koller, Angela Koutsokera, Andreas Kremer, Thorsten Krueger, Christian Kuhn, Arnaud L’Huillier, Bettina Laesser, Frédéric Lamoth, Roger Lehmann, Alexander Leichtle, Oriol Manuel, Hans-Peter Marti, Michele Martinelli, Valérie McLin, Katell Mellac, Aurélia Merçay, Karin Mettler, Sara Christina Meyer, Nicolas Müller, Jelena Müller, Ulrike Müller-Arndt, Mirjam Nägeli, Dionysios Neofytos, Jakob Nilsson, Manuel Pascual, Rosmarie Pazeller, David Reineke, Juliane Rick, Fabian Rössler, Silvia Rothlin, Thomas Schachtner, Stefan Schaub, Dominik Schneidawind, Macé Schuurmans, Simon Schwab, Thierry Sengstag, Daniel Sidler, Federico Simonetta, Jürg Steiger, Guido Stirnimann, Ueli Stürzinger, Christian Van Delden, Jean-Pierre Venetz, Jean Villard, Julien Vionnet, Laura Walti, Caroline Wehmeier, Patrick Yerly.
