# Supplementary material for: Burden of infectious diseases in children during the first year after solid organ transplantation
Source: Transpl Int. 2026 May 22;39:16075. doi: 10.3389/ti.2026.16075 (PMC13240429; doi:10.3389/ti.2026.16075)
Supplement: Supplementary file 1 [file DataSheet1.pdf]

Transplanted organ	Kidney	Liver	Lung	Heart	Overall*
Number of viral IDE	n = 51	n = 113	n = 11	n = 14	n = 189
**Virus, n (%)**					
Adenovirus	0 (0.0)	14 (12.4)	0 (0.0)	2 (14.3)	16 (8.5)
BK virus	10 (19.6)	0 (0.0)	0 (0.0)	0 (0.0)	10 (5.3)
Cytomegalovirus	12 (23.5)	9 (8.0)	2 (18.2)	1 (7.1)	24 (12.7)
Coronaviruses (SARS-CoV-2 and HCoVs)	4 (7.8)	11 (9.7)	1 (9.1)	1 (7.1)	17 (9.0)
Epstein-Barr virus	0 (0.0)	8 (7.1)	0 (0.0)	0 (0.0)	8 (4.2)
Hepatitis B virus	0 (0.0)	1 (0.9)	0 (0.0)	0 (0.0)	1 (0.5)
Human herpesvirus type 6	0 (0.0)	13 (11.5)	0 (0.0)	0 (0.0)	13 (6.9)
Herpes simplex virus	3 (5.9)	0 (0.0)	0 (0.0)	1 (7.1)	4 (2.1)
Influenza	3 (5.9)	3 (2.7)	0 (0.0)	1 (7.1)	7 (3.7)
Norovirus	2 (3.9)	9 (8.0)	0 (0.0)	1 (7.1)	12 (6.3)
Parainfluenza	2 (3.9)	1 (0.9)	0 (0.0)	0 (0.0)	3 (1.6)
Picornaviruses	3 (5.9)	6 (5.3)	6 (54.5)	3 (21.4)	18 (9.5)
Rotavirus	2 (3.9)	8 (7.1)	0 (0.0)	2 (14.3)	12 (6.3)
Respiratory syncytial virus	1 (2.0)	2 (1.8)	1 (9.1)	0 (0.0)	4 (2.1)
Varicella-zoster virus	3 (5.9)	1 (0.9)	0 (0.0)	2 (14.3)	6 (3.2)
Other respiratory viruses	1 (2.0)	10 (8.8)	1 (9.1)	0 (0.0)	12 (6.3)
Other viruses	4 (7.8)	5 (4.4)	0 (0.0)	0 (0.0)	9 (4.8)
*Multiple viruses***	1 (2.0)	12 (10.6)	0 (0.0)	0 (0.0)	13 (6.9)

Transplanted organ	Kidney	Liver	Lung	Heart	Overall*
Number of bacterial IDE	n = 60	n = 62	n = 12	n = 14	n = 149
**Bacteria, n (%)**					
Acinetobacter spp	0 (0.0)	0 (0.0)	1 (8.3)	0 (0.0)	1 (0.7)
Clostridium spp	8 (13.3)	5 (8.1)	2 (16.7)	4 (28.6)	19 (12.8)
Coagulase negative staphylococci	4 (6.7)	9 (14.5)	0 (0.0)	1 (7.1)	14 (9.4)
E. coli	17 (28.3)	6 (9.7)	0 (0.0)	2 (14.3)	25 (16.8)
Enterobacter spp	3 (5.0)	1 (1.6)	0 (0.0)	2 (14.3)	6 (4.0)
Enterococcus spp	1 (1.7)	5 (8.1)	3 (25.0)	1 (7.1)	10 (6.7)
Klebsiella spp	8 (13.3)	1 (1.6)	0 (0.0)	0 (0.0)	9 (6.0)
Miscellaneous bacteria	3 (5.0)	0 (0.0)	1 (8.3)	1 (7.1)	5 (3.4)
Nontuberculosis mycobacteria	0 (0.0)	0 (0.0)	2 (16.7)	0 (0.0)	2 (1.3)
Pseudomonas spp	7 (11.7)	10 (16.1)	1 (8.3)	1 (7.1)	19 (12.8)
S. aureus	0 (0.0)	1 (1.6)	0 (0.0)	2 (14.3)	3 (2.0)
Stenotrophomonas spp	0 (0.0)	2 (3.2)	1 (8.3)	0 (0.0)	3 (2.0)
Streptococcus spp	1 (1.7)	4 (6.5)	0 (0.0)	0 (0.0)	5 (3.4)
Other anaerobic bacteria	0 (0.0)	1 (1.6)	0 (0.0)	0 (0.0)	1 (0.7)
Other enterobacteriaceae	4 (6.7)	1 (1.6)	0 (0.0)	0 (0.0)	5 (3.4)
Other gram-negative bacteria	1 (1.7)	2 (3.2)	0 (0.0)	0 (0.0)	3 (2.0)
Other gram-positive bacteria	0 (0.0)	1 (1.6)	0 (0.0)	0 (0.0)	1 (0.7)
Multiple bacteria	3 (5.0)	13 (21.0)	1 (8.3)	0 (0.0)	18 (12.1)

Transplanted organ	Kidney	Liver	Lung	Heart	Overall*
Number of fungal IDE	n = 0	n = 16	n = 3	n = 3	n = 22
**Fungi, n (%)**					
Aspergillus spp	0 (NA)	2 (12.5)	1 (33.3)	0 (0.0)	3 (13.6)
Candida albicans	0 (NA)	10 (62.5)	1 (33.3)	2 (66.7)	13 (59.1)
Pneumocystis spp	0 (NA)	0 (0.0)	0 (0.0)	1 (33.3)	1 (4.5)
Other candida spp	0 (NA)	4 (25.0)	1 (33.3)	0 (0.0)	5 (22.7)

HCoVs: human coronaviruses; IDE: infectious diseases event

* including 1 IDE from combined transplant non displayed in the table

*including 2 concomitant EBV-CMV infections; 2 EBV/HHV6 co-infections and 1 CMV HHV6 co-infections

Supplementary table 1a

**Age group**	**0-0.99 vr**	**1-4.99 yrs**	**5-11.99 yrs**	**12-17.99 yrs**	**Overall**
Number of viral IDE	n = 93	n = 24	n = 32	n = 40	n = 189
**Virus, n (%)**					
Adenovirus	11 (11.8)	3 (12.5)	0 (0.0)	2 (5.0)	16 (8.5)
BK virus	0 (0.0)	4 (16.7)	2 (6.3)	4 (10.0)	10 (5.3)
Cytomegalovirus	10 (10.8)	2 (8.3)	4 (12.5)	8 (20.0)	24 (12.7)
Coronaviruses (SARS-CoV-2 and HCoVs)	9 (9.7)	1 (4.2)	2 (6.3)	5 (12.5)	17 (9.0)
Epstein-Barr virus	5 (5.4)	1 (4.2)	0 (0.0)	2 (5.0)	8 (4.2)
Hepatitis B virus	0 (0.0)	0 (0.0)	1 (3.1)	0 (0.0)	1 (0.5)
Human herpesvirus type 6	9 (9.7)	2 (8.3)	1 (3.1)	1 (2.5)	13 (6.9)
Herpes simplex virus	0 (0.0)	0 (0.0)	2 (6.3)	2 (5.0)	4 (2.1)
Influenza	2 (2.2)	0 (0.0)	1 (3.1)	4 (10.0)	7 (3.7)
Norovirus	9 (9.7)	2 (8.3)	1 (3.1)	0 (0.0)	12 (6.3)
Parainfluenza	1 (1.1)	1 (4.2)	1 (3.1)	0 (0.0)	3 (1.6)
Picornaviruses	7 (7.5)	1 (4.2)	6 (18.8)	4 (10.0)	18 (9.5)
Rotavirus	7 (7.5)	2 (8.3)	2 (6.3)	1 (2.5)	12 (6.3)
Respiratory syncytial virus	2 (2.2)	1 (4.2)	1 (3.1)	0 (0.0)	4 (2.1)
Varicella-zoster virus	1 (1.1)	1 (4.2)	0 (0.0)	4 (10.0)	6 (3.2)
Other respiratory viruses	6 (6.5)	1 (4.2)	3 (9.4)	2 (5.0)	12 (6.3)
Other viruses	4 (4.3)	0 (0.0)	5 (15.6)	0 (0.0)	9 (4.8)
*Multiple viruses**	10 (10.8)	2 (8.3)	0 (0.0)	1 (2.5)	13 (6.9)

**Age group**	**0-0.99 yr**	**1-4.99 yrs**	**5-11.99 yrs**	**12-17.99 yrs**	**Overall**
Number of bacterial IDE	n = 70	n = 18	n = 14	n = 47	n = 149
**Bacteria, n (%)**					
Acinetobacter spp	0 (0.0)	0 (0.0)	0 (0.0)	1 (2.1)	1 (0.7)
Clostridium spp	9 (12.9)	3 (16.7)	2 (14.3)	5 (10.6)	19 (12.8)
Coagulase negative staphylococci	10 (14.3)	0 (0.0)	2 (14.3)	2 (4.3)	14 (9.4)
E. coli	5 (7.1)	6 (33.3)	4 (28.6)	10 (21.3)	25 (16.8)
Enterobacter spp	5 (7.1)	0 (0.0)	0 (0.0)	1 (2.1)	6 (4.0)
Enterococcus spp	3 (4.3)	0 (0.0)	1 (7.1)	6 (12.8)	10 (6.7)
Klebsiella spp	6 (8.6)	2 (11.1)	0 (0.0)	1 (2.1)	9 (6.0)
Miscellaneous bacteria	0 (0.0)	0 (0.0)	0 (0.0)	5 (10.6)	5 (3.4)
Nontuberculosis mycobacteria	0 (0.0)	0 (0.0)	1 (7.1)	1 (2.1)	2 (1.3)
Pseudomonas spp	9 (12.9)	3 (16.7)	2 (14.3)	5 (10.6)	19 (12.8)
S. aureus	1 (1.4)	0 (0.0)	1 (7.1)	1 (2.1)	3 (2.0)
Stenotrophomonas spp	1 (1.4)	1 (5.6)	1 (7.1)	0 (0.0)	3 (2.0)
Streptococcus spp	3 (4.3)	1 (5.6)	0 (0.0)	1 (2.1)	5 (3.4)
Other anaerobic bacteria	1 (1.4)	0 (0.0)	0 (0.0)	0 (0.0)	1 (0.7)
Other enterobacteriaceae	5 (7.1)	0 (0.0)	0 (0.0)	0 (0.0)	5 (3.4)
Other gram-negative bacteria	1 (1.4)	0 (0.0)	0 (0.0)	2 (4.3)	3 (2.0)
Other gram-positive bacteria	1 (1.4)	0 (0.0)	0 (0.0)	0 (0.0)	1 (0.7)
Multiple bacteria	10 (14.3)	2 (11.1)	0 (0.0)	6 (12.8)	18 (12.1)

**Age group**	**0-0.99 yrs**	**1-4.99 yrs**	**5-11.99 yrs**	**12-17.99 yrs**	**Overall**
Number of fungal IDE	n = 9	n = 2	n = 1	n = 10	n = 22
**Fungi, n (%)**					
Aspergillus spp	0 (0.0)	0 (0.0)	0 (0.0)	3 (30.0)	3 (13.6)
Candida albicans	5 (55.6)	2 (100.0)	1 (100.0)	5 (50.0)	13 (59.1)
Pneumocystis spp	1 (11.1)	0 (0.0)	0 (0.0)	0 (0.0)	1 (4.5)
Other candida spp	3 (33.3)	0 (0.0)	0 (0.0)	2 (20.0)	5 (22.7)

HCoVs: human coronaviruses; IDE: infectious diseases event; yr: year
**including 2 concomitant EBV-CMV infections; 2 EBV/HHV6 co-infections and 1 CMV HHV6 co-infections

Supplementary table 1b

Transplanted organ	Kidney	Liver	Lung	Heart	Overall*
Number of viral IDE	n = 51	n = 113	n = 11	n= 14	n = 189
**Virus, n (%)**					
Blood	13 (25.5)	20 (17.7)	1 (9.1)	0 (0.0)	34 (18.0)
Central nervous system	0 (0.0)	0 (0.0)	0 (0.0)	1 (7.1)	1 (0.5)
Gastro-intestinal	5 (9.8)	38 (33.6)	1 (9.1)	6 (42.9)	50 (26.5)
Liver	0 (0.0)	4 (3.5)	0 (0.0)	0 (0.0)	4 (2.1)
Mucocutaneous	8 (15.7)	1 (0.9)	0 (0.0)	2 (14.3)	11 (5.8)
Multiple infection sites	4 (7.8)	16 (14.2)	1 (9.1)	0 (0.0)	21 (11.1)
Other/unidentified	1 (2.0)	1 (0.9)	0 (0.0)	0 (0.0)	2 (1.1)
Respiratory tract	17 (33.3)	33 (29.2)	8 (72.7)	5 (35.7)	63 (33.3)
Urinary tract	3 (5.9)	0 (0.0)	0 (0.0)	0 (0.0)	3 (1.6)

Transplanted organ	Kidney	Liver	Lung	Heart	Overall*
Number of bacterial IDE	n = 60	n = 62	n = 12	n = 14	n = 149
**Bacteria, n (%)**					
Blood	3 (5.0)	7 (11.3)	0 (0.0)	2 (14.3)	12 (8.1)
Catheter	1 (1.7)	4 (6.5)	0 (0.0)	0 (0.0)	5 (3.4)
Ear-nose-throat	0 (0.0)	1 (1.6)	0 (0.0)	0 (0.0)	1 (0.7)
Gastro-intestinal	8 (13.3)	13 (21.0)	2 (16.7)	4 (28.6)	28 (18.8)
Liver	0 (0.0)	8 (12.9)	0 (0.0)	0 (0.0)	8 (5.4)
Mucocutaneous	4 (6.7)	1 (1.6)	0 (0.0)	2 (14.3)	7 (4.7)
Multiple infection sites	2 (3.3)	17 (27.4)	3 (25.0)	1 (7.1)	23 (15.4)
Other/unidentified	0 (0.0)	2 (3.2)	0 (0.0)	0 (0.0)	2 (1.3)
Preservation liquid	0 (0.0)	1 (1.6)	0 (0.0)	0 (0.0)	1 (0.7)
Prosthetic	1 (1.7)	0 (0.0)	0 (0.0)	0 (0.0)	1 (0.7)
Respiratory tract	0 (0.0)	4 (6.5)	7 (58.3)	3 (21.4)	14 (9.4)
Surgical site infection	1 (1.7)	1 (1.6)	0 (0.0)	1 (7.1)	3 (2.0)
Urinary tract	40 (66.7)	3 (4.8)	0 (0.0)	1 (7.1)	44 (29.5)

Transplanted organ	Kidney	Liver	Lung	Heart	Overall*
Number of fungal IDE	n = 0	n = 16	n = 3	n = 3	n = 22
**Fungi, n (%)**					
Blood	0 (NA)	0 (0.0)	1 (33.3)	0 (0.0)	1 (4.5)
Ear-nose-throat	0 (NA)	0 (0.0)	0 (0.0)	1 (33.3)	1 (4.5)
Gastro-intestinal	0 (NA)	2 (12.5)	0 (0.0)	0 (0.0)	2 (9.1)
Liver	0 (NA)	1 (6.3)	0 (0.0)	0 (0.0)	1 (4.5)
Mucocutaneous	0 (NA)	5 (31.3)	0 (0.0)	0 (0.0)	5 (22.7)
Multiple infection sites	0 (NA)	5 (31.3)	1 (33.3)	0 (0.0)	6 (27.3)
Other/unidentified	0 (NA)	0 (0.0)	0 (0.0)	1 (33.3)	1 (4.5)
Preservation liquid	0 (NA)	1 (6.3)	0 (0.0)	0 (0.0)	1 (4.5)
Respiratory tract	0 (NA)	1 (6.3)	1 (33.3)	1 (33.3)	3 (13.6)
Surgical site infection	0 (NA)	1 (6.3)	0 (0.0)	0 (0.0)	1 (4.5)

IDE: infectious diseases event

* including 1 IDE from combined transplant non displayed in the table

Supplementary table 2

**Time after transplantation**<br>Number of viral IDE	**0-1 month**<br>n= 41	**1-3 months**<br>n = 34	**3-6 months**<br>n = 46	**6-12 months**<br>n = 68	**Overall**<br>n = 189
**Virus, n (%)**					
Adenovirus	3 (7.3)	1 (2.9)	6 (13.0)	6 (8.8)	16 (8.5)
BK virus	0 (0.0)	5 (14.7)	3 (6.5)	2 (2.9)	10 (5.3)
Cytomegalovirus	5 (12.2)	7 (20.6)	2 (4.3)	10 (14.7)	24 (12.7)
Coronaviruses (SARS-CoV-2 and HCoVs)	2 (4.9)	1 (2.9)	5 (10.9)	9 (13.2)	17 (9.0)
Epstein-Barr virus	0 (0.0)	1 (2.9)	6 (13.0)	1 (1.5)	8 (4.2)
Hepatitis B virus	1 (2.4)	0 (0.0)	0 (0.0)	0 (0.0)	1 (0.5)
Human herpesvirus type 6	7 (17.1)	1 (2.9)	1 (2.2)	4 (5.9)	13 (6.9)
Herpes simplex virus	2 (4.9)	1 (2.9)	0 (0.0)	1 (1.5)	4 (2.1)
Influenza	0 (0.0)	0 (0.0)	3 (6.5)	4 (5.9)	7 (3.7)
Norovirus	2 (4.9)	2 (5.9)	4 (8.7)	4 (5.9)	12 (6.3)
Parainfluenza	0 (0.0)	0 (0.0)	0 (0.0)	3 (4.4)	3 (1.6)
Rhino/enteroviruses	3 (7.3)	3 (8.8)	7 (15.2)	5 (7.4)	18 (9.5)
Rotavirus	3 (7.3)	3 (8.8)	3 (6.5)	3 (4.4)	12 (6.3)
Respiratory syncytial virus	2 (4.9)	0 (0.0)	1 (2.2)	1 (1.5)	4 (2.1)
Varicella-zoster virus	1 (2.4)	2 (5.9)	1 (2.2)	2 (2.9)	6 (3.2)
Other respiratory viruses	3 (7.3)	4 (11.8)	1 (2.2)	4 (5.9)	12 (6.3)
Other viruses	2 (4.9)	2 (5.9)	0 (0.0)	5 (7.4)	9 (4.8)
*Multiple viruses**	5 (12.2)	1 (2.9)	3 (6.5)	4 (5.9)	13 (6.9)

**Time after transplantation**<br>Number of bacterial IDE	**0-1 months**<br>n = 49	**1-3 months**<br>n = 35	**3-6 months**<br>n = 17	**6-12 months**<br>n = 48	**Overall**<br>n = 149
**Bacteria, n (%)**					
Acinetobacter spp	1 (2.0)	0 (0.0)	0 (0.0)	0 (0.0)	1 (0.7)
Clostridium spp	5 (10.2)	8 (22.9)	2 (11.8)	4 (8.3)	19 (12.8)
Coagulase negative staphylococci	7 (14.3)	1 (2.9)	1 (5.9)	5 (10.4)	14 (9.4)
E. coli	9 (18.4)	4 (11.4)	4 (23.5)	8 (16.7)	25 (16.8)
Enterobacter spp	3 (6.1)	0 (0.0)	2 (11.8)	1 (2.1)	6 (4.0)
Enterococcus spp	3 (6.1)	3 (8.6)	0 (0.0)	4 (8.3)	10 (6.7)
Klebsiella spp	0 (0.0)	1 (2.9)	1 (5.9)	7 (14.6)	9 (6.0)
Miscellaneous bacteria	1 (2.0)	1 (2.9)	1 (5.9)	2 (4.2)	5 (3.4)
Nontuberculosis mycobacteria	2 (4.1)	0 (0.0)	0 (0.0)	0 (0.0)	2 (1.3)
Pseudomonas spp	7 (14.3)	5 (14.3)	1 (5.9)	6 (12.5)	19 (12.8)
S. aureus	0 (0.0)	1 (2.9)	1 (5.9)	1 (2.1)	3 (2.0)
Stenotrophomonas spp	3 (6.1)	0 (0.0)	0 (0.0)	0 (0.0)	3 (2.0)
Streptococcus spp	2 (4.1)	1 (2.9)	0 (0.0)	2 (4.2)	5 (3.4)
Other anaerobic bacteria	0 (0.0)	1 (2.9)	0 (0.0)	0 (0.0)	1 (0.7)
Other enterobacteriaceae	0 (0.0)	1 (2.9)	0 (0.0)	4 (8.3)	5 (3.4)
Other gram-negative bacteria	0 (0.0)	1 (2.9)	1 (5.9)	1 (2.1)	3 (2.0)
Other gram-positive bacteria	0 (0.0)	1 (2.9)	0 (0.0)	0 (0.0)	1 (0.7)
Multiple bacteria	6 (12.2)	6 (17.1)	3 (17.6)	3 (6.3)	18 (12.1)

**Time after transplantation**<br>Number of fungal IDE	**0-1 month**<br>n = 14	**1-3 months**<br>n = 3	**3-6 months**<br>n = 2	**6-12 months**<br>n = 3	**Overall**<br>n = 22
**Fungi, n (%)**					
Aspergillus spp	2 (14.3)	0 (0.0)	1 (50.0)	0 (0.0)	3 (13.6)
Candida albicans	8 (57.1)	2 (66.7)	1 (50.0)	2 (66.7)	13 (59.1)
Pneumocystis spp	1 (7.1)	0 (0.0)	0 (0.0)	0 (0.0)	1 (4.5)
Other candida spp	3 (21.4)	1 (33.3)	0 (0.0)	1 (33.3)	5 (22.7)

HCoVs: human coronaviruses; IDE: infectious diseases event
*including 2 concomitant EBV-CMV infections; 2 EBV/HHV6 co-infections and 1 CMV HHV6 co-infections

Supplementary table 3

Transplanted organ<br>Number of rejection episodes	Kidney<br>n = 34[1]	Liver<br>n = 36[1]	Lung<br>n = 2[1]	Heart<br>n = 82[1]	Overall*<br>n = 155[1]
Median time between SOT and rejection, days (IQR)	75 (11, 195)	40 (13, 142)	307 (252, 361)	48 (21, 127)	51 (18, 166)
**IDE between SOT and rejection**					
All IDE	6 (18%)	16 (44%)	1 (50%)	35 (43%)	58 (37%)
within 30 days before rejectio	*2 (5.9%)*	*11 (31%)*	*0 (0%)*	*11 (13%)*	*24 (15%)*
CMV event	4 (12%)	2 (5.6%)	0 (0%)	2 (2.4%)	8 (5.2%)
within 30 days before rejection	*0 (0%)*	*1 (2.8%)*	*0 (0%)*	*0 (0%)*	*1 (0.6%)*
**IDE in the next 90 days following rejection**					
0	27 (79%)	25 (69%)	2 (100%)	75 (91%)	130 (84%)
1	6 (18%)	5 (14%)	0 (0%)	5 (6.1%)	16 (10%)
>1	1 (2.9%)	6 (16.8%)	0 (0%)	2 (2.4%)	9 (5.8%)

[1]n (%); Median (Q1, Q3)

SOT: solid organ transplantation; IDE: infectious disease event; CMV: cytomegalovirus; IQR: interquartile range

* including 1 IDE from combined transplant non displayed in the table

Supplementary table 4

Supplementary Figure 1. Study flowchart

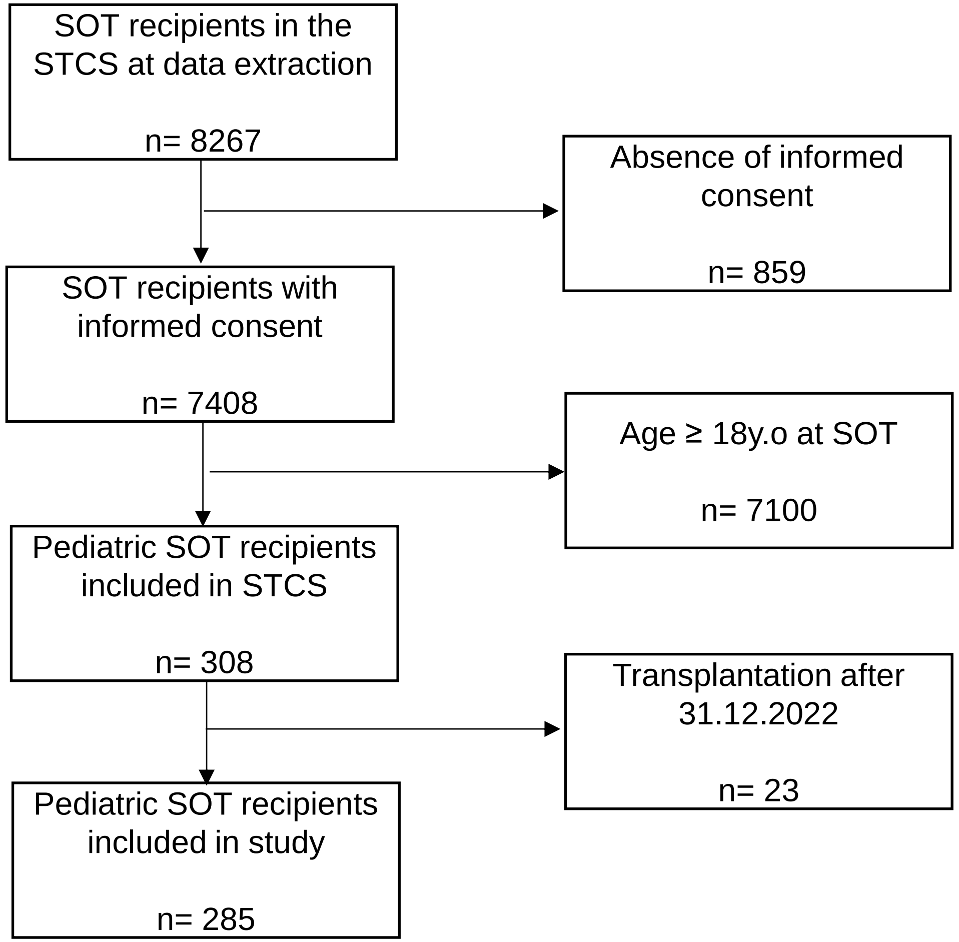

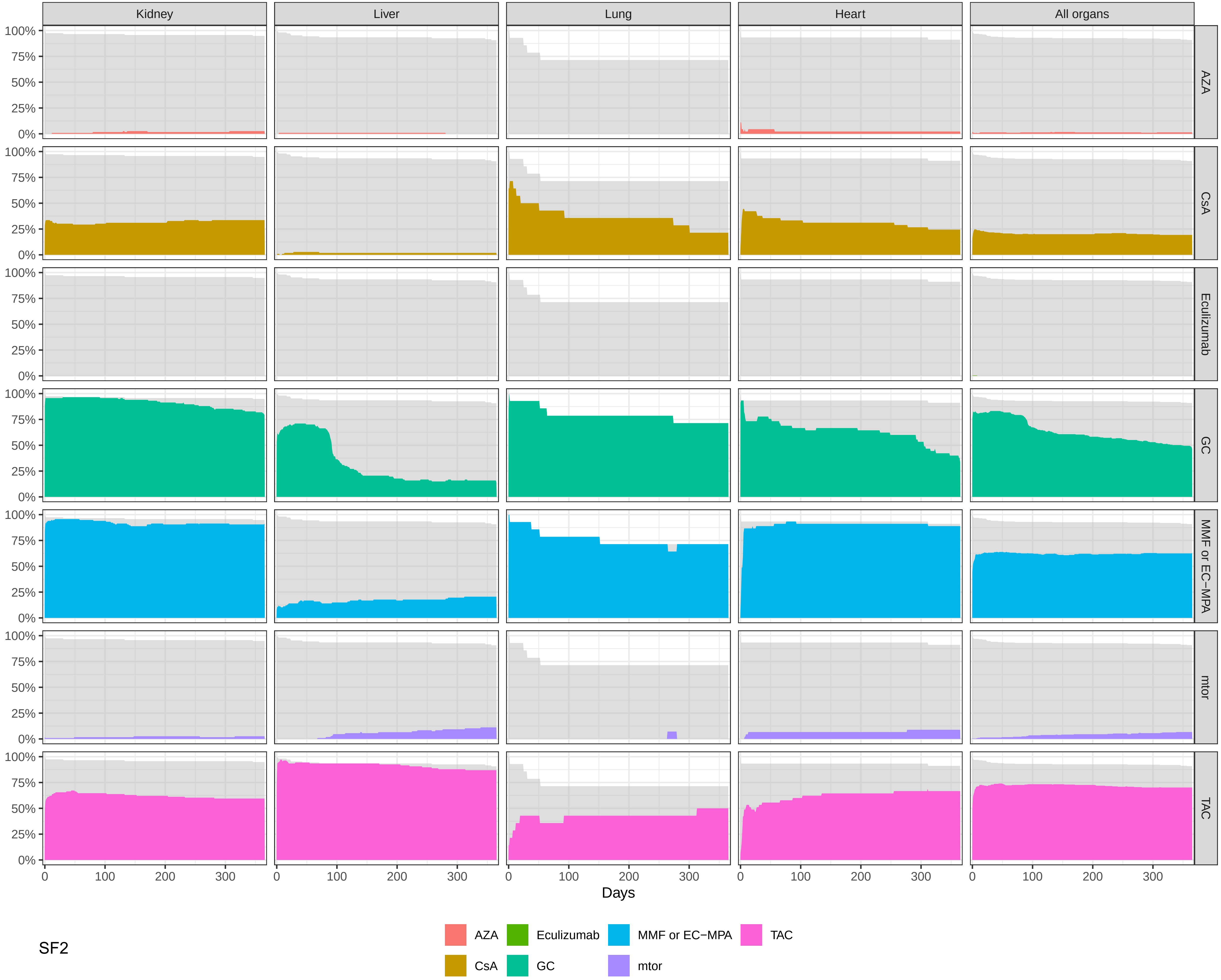
Kidney
Liver
Lung
Heart
All organs
AZA
CsA
Eculizumab
GC
MMF or EC−MPA
mtor
TAC
100%
75%
50%
25%
0%
0
100
200
300
Days
AZA
Eculizumab
MMF or EC−MPA
TAC
CsA
GC
mtor

SF2

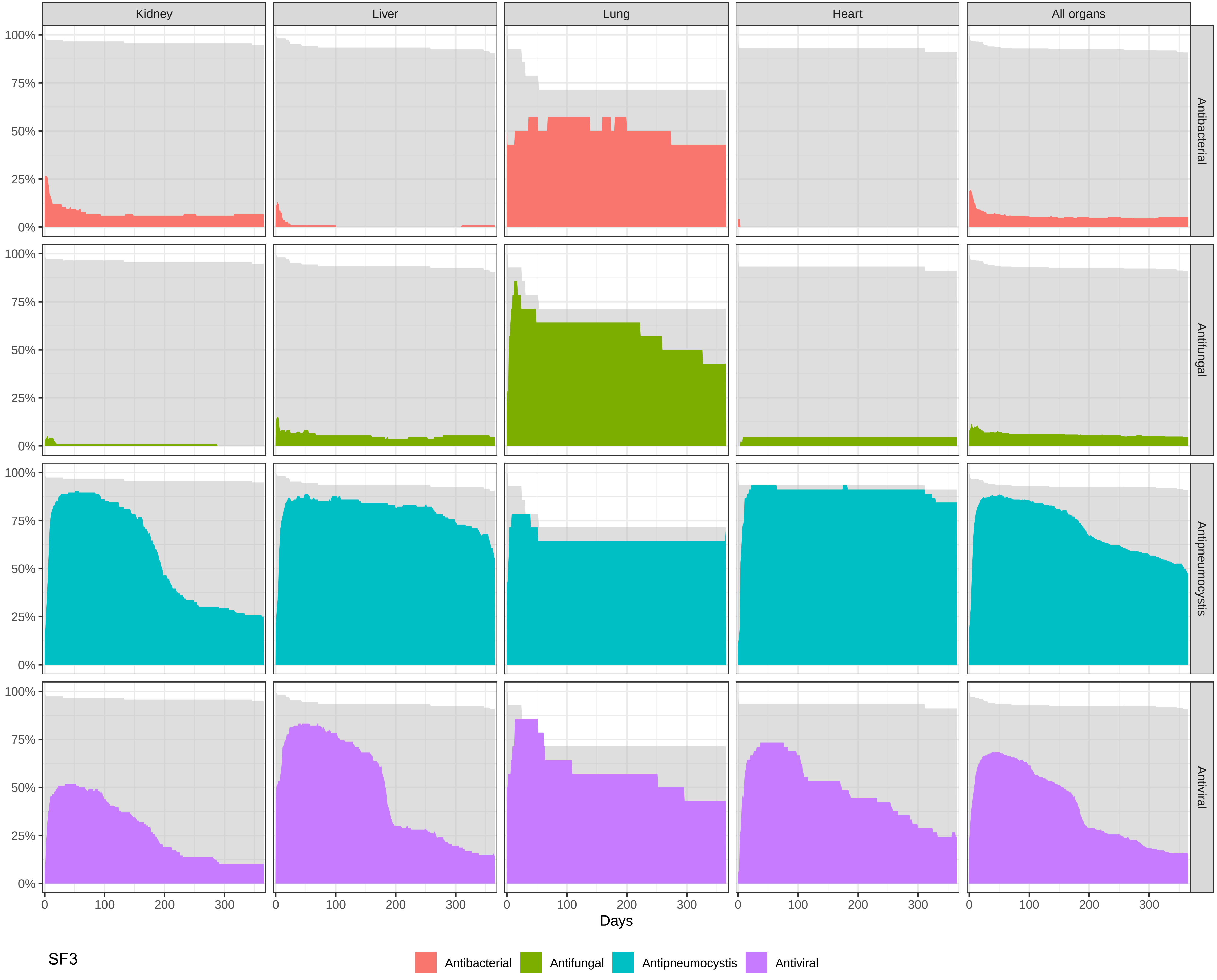
Kidney
Liver
Lung
Heart
All organs
Antibacterial
Antifungal
Antipneumocystis
Antiviral
100%
75%
50%
25%
0%
0
100
200
300
Days
Antibacterial
Antifungal
Antipneumocystis
Antiviral

SF3

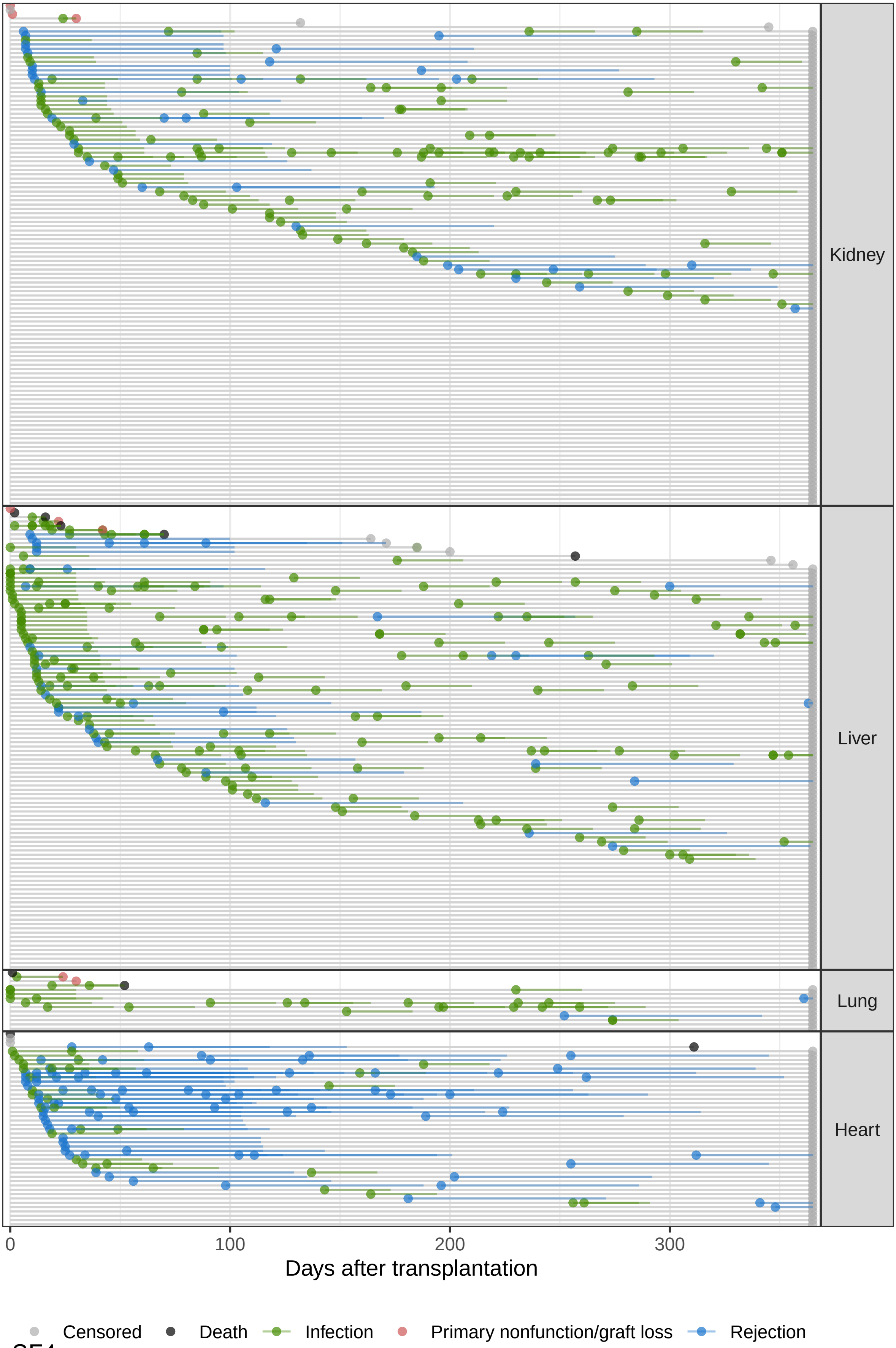

Kidney
Liver
Lung
Heart
0
100
200
300
Days after transplantation
Censored
Death
Infection
Primary nonfunction/graft loss
Rejection

SF4

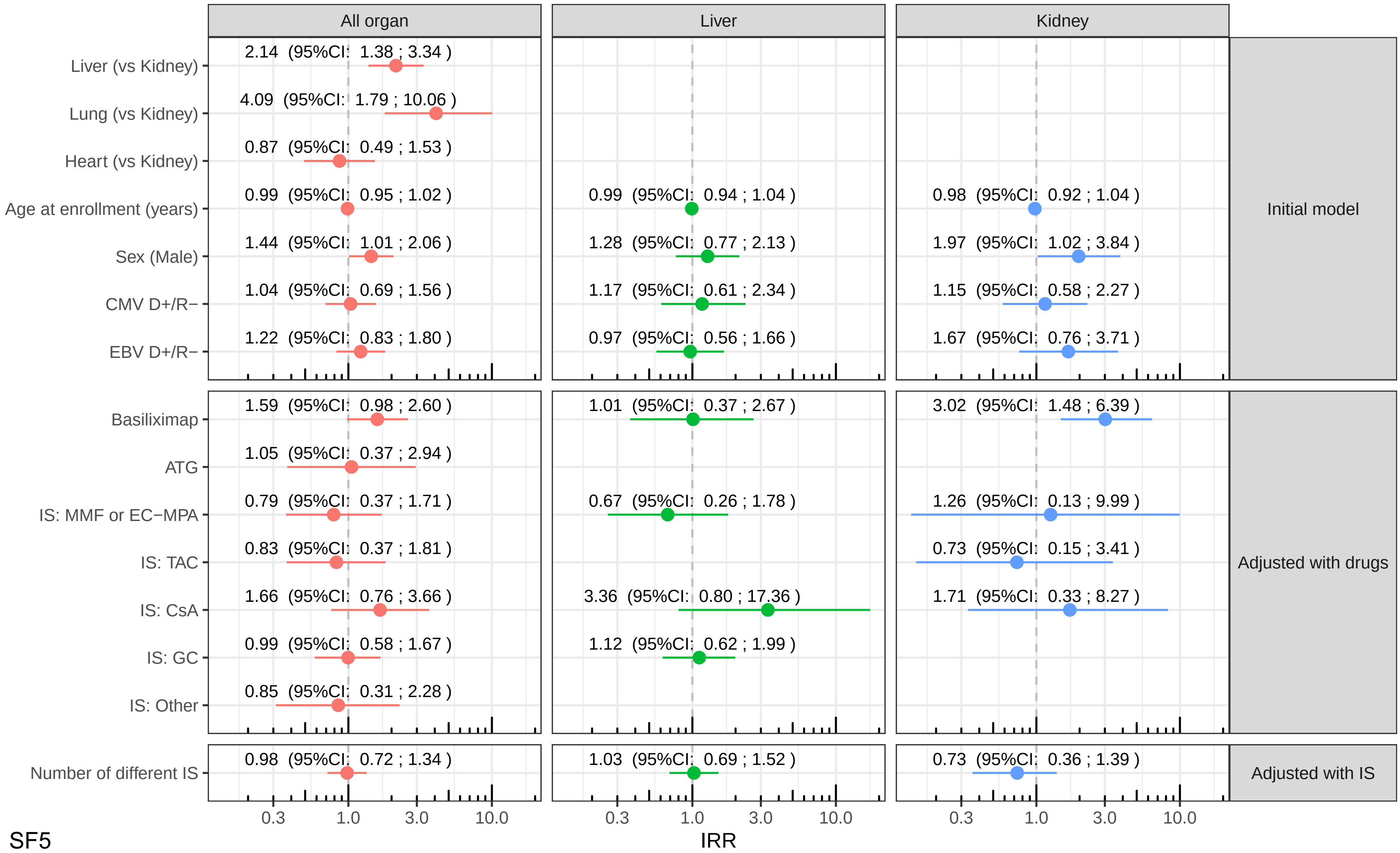
All organ
Liver
Kidney
Liver (vs Kidney)
Lung (vs Kidney)
Heart (vs Kidney)
Age at enrollment (years)
Sex (Male)
CMV D+/R−
EBV D+/R−
Basiliximap
ATG
IS: MMF or EC−MPA
IS: TAC
IS: CsA
IS: GC
IS: Other
Number of different IS
2.14 (95%CI: 1.38 ; 3.34)
4.09 (95%CI: 1.79 ; 10.06)
0.87 (95%CI: 0.49 ; 1.53)
0.99 (95%CI: 0.95 ; 1.02)
1.44 (95%CI: 1.01 ; 2.06)
1.04 (95%CI: 0.69 ; 1.56)
1.22 (95%CI: 0.83 ; 1.80)
0.99 (95%CI: 0.94 ; 1.04)
1.28 (95%CI: 0.77 ; 2.13)
1.17 (95%CI: 0.61 ; 2.34)
0.97 (95%CI: 0.56 ; 1.66)
0.98 (95%CI: 0.92 ; 1.04)
1.97 (95%CI: 1.02 ; 3.84)
1.15 (95%CI: 0.58 ; 2.27)
1.67 (95%CI: 0.76 ; 3.71)
Initial model
1.59 (95%CI: 0.98 ; 2.60)
1.05 (95%CI: 0.37 ; 2.94)
0.79 (95%CI: 0.37 ; 1.71)
0.83 (95%CI: 0.37 ; 1.81)
1.66 (95%CI: 0.76 ; 3.66)
0.99 (95%CI: 0.58 ; 1.67)
0.85 (95%CI: 0.31 ; 2.28)
1.01 (95%CI: 0.37 ; 2.67)
0.67 (95%CI: 0.26 ; 1.78)
3.36 (95%CI: 0.80 ; 17.36)
1.12 (95%CI: 0.62 ; 1.99)
3.02 (95%CI: 1.48 ; 6.39)
1.26 (95%CI: 0.13 ; 9.99)
0.73 (95%CI: 0.15 ; 3.41)
1.71 (95%CI: 0.33 ; 8.27)
Adjusted with drugs
0.98 (95%CI: 0.72 ; 1.34)
1.03 (95%CI: 0.69 ; 1.52)
0.73 (95%CI: 0.36 ; 1.39)
Adjusted with IS
0.3
1.0
3.0
10.0
IRR
SF5

Supplementary file 1: Detailed statistical method

Baseline characteristics of donors and recipients were described using count and percentages for categorical data, and median and interquartile range (IQR) for continuous variables. Cumulative incidence of the first infection, with death, graft failure or second transplant defined as competing risks, were calculated by organs and by age groups; the Gray tests comparing the sub-groups were reported accordingly (15). Rate of IDE expressed as IDE/person/year (IDE/p/y) was computed using Poisson generalized linear model. Negative binomial generalized linear models (GLM), adjusted for the follow-up time, were employed to assess the risk factor of clinically relevant infections during the first-year post-transplantation. As patients age, induction and maintenance immunosuppression differ across organs, we performed several multivariable models. The first GLM included baseline risk factor selected a priori such as sex, age, EBV and CMV risk stratification and organ type which we defined as the set of variables RF1. The second GLM included organ-dependent variables such as induction and immunosuppression regimens in addition to the variable RF1. Moreover, a third GLM included the induction and the number of different immunosuppressive drugs in addition to the variable RF1. All risk factors were also assessed individually in univariable analyses. Finally, sensitivity analysis were conducted separately for liver and kidney recipients using the same modeling approach.

Moreover, two cause-specific hazard models, with death, graft failure or second transplant defined as competing risks, and stratified by organs were used to investigate the link between rejection and IDE, and were expressed using hazard ratio (HR); the first assessed the risk of clinically relevant infections within 90 days of a documented episode of rejection, defined as a time-dependent covariate, and the second assessed the risk of rejection within 30 days of a documented infectious event, defined as a time-dependent covariate. Both models included the recipient sex, CMV and EBV risk stratification as well as age as baseline covariates. Infections and rejections, along with the competing risks, were represented as scatter plot over time for each patient, stratified by organ and included a predefined 90-days risk period post-rejection and 30-days risk period post-infection as well. R 4.4.0 was used to perform the statistical analysis (16). Data manipulation and visualization was made using the tidyverse suite (17), survival analysis was performed using the survival R package (18) and negative binomial generalized linear models were fitted using the MASS R package(19).
